# Psychological stress and influencing factors of hospital workers in different periods under the public health background of infectious disease outbreak: A cross‐sectional study

**DOI:** 10.1002/hsr2.834

**Published:** 2022-09-15

**Authors:** Xing Miao, Wei Wang, Yongli Chen, Xiufang Huang, Rehua Wang

**Affiliations:** ^1^ Department of Cardiology Fujian Provincial Hospital Fuzhou China; ^2^ Department of Cardiology Shengli Clinical Medical College of Fujian Medical University Fuzhou China; ^3^ Department of Psychiatry Fuzhou Neuropsychiatric Hospital Affiliated to Fujian Medical University Fuzhou China

**Keywords:** anxiety, depression, infectious disease outbreak, medical workers

## Abstract

**Background and Aims:**

It is well known that public health emergencies can affect the mental health of medical personnel, and many studies have focused on cross‐sectional studies with short‐term benefits. The present study aimed to investigate the long‐term influence of infectious disease outbreak about the mental health of hospital staff.

**Methods:**

The demographic characteristics and mental health status of staff in Fuzhou, China, were analyzed by using the Generalized Anxiety Disorder (GAD‐7) Scale and Depression Screening Scale (9‐item Patient Health Questionnaire [PHQ‐9]) in February and December 2020.

**Results:**

There were no significant differences in anxiety levels during different time periods (*p* > 0.05), but there were significant differences among anxiety level and total score of GAD‐7 scale (*p* < 0.001). There were significant differences among the number of people with depression, depression level, and total score on the PHQ‐9 scale (*p* < 0.001). As the pandemic progressed, total scores of anxiety in medical staff with different titles decreased (*p* < 0.05), but depression scores in professionals with intermediate and senior titles increased significantly (*p* < 0.05). changes in anxiety and depression scores during different time periods also changed according to hospital worker specialty. Total scores of anxiety in doctors, nurses, medical technicians, and other staff members all decreased (*p* < 0.05), while total scores of depression in doctors, nurses, and other staff members significantly increased (*p* < 0.05). There were no significant differences in total depression score among medical technicians (*p* > 0.05).

**Conclusions:**

Since the outbreak of an infectious disease public health emergency, the anxiety of hospital staff has decreased over time, but the depression has increased. The management and psychological support personnel in medical institutions should continue to pay attention to the mental health of medical staff, and it is necessary to take different intervention measures in different periods when implementing the psychological crisis prevention mechanism.

## 1 INTRODUCTION

A public health emergency is a unique event that poses a serious threat to human health, causes catastrophic economic losses, and can even cause mass panic. Novel coronavirus disease (COVID‐19) is the most serious infectious disease pandemic in 100 years and was defined as a major health emergency by WHO on 30 January 2020.^1^ It has been linked to severe acute respiratory syndrome (SARS) in 2003, the H1N1 influenza virus subtype in 2009, Middle East respiratory syndrome (MERS) in 2012, and Ebola virus disease in 2014.^2^, ^3^, ^4^ It has brought serious psychological stress reaction and psychological disorder to all social groups.^5^

Medical staff, as a special group, are more prone to psychological abnormalities such as anxiety and depression than ordinary people.

The main source for its spread in hospitals is among hospital workers, the main professionals dealing with this health emergency who are under pressure to treat patients and are at great risk of infection.^6^ Therefore, psychological stress monitoring and supervision of hospital staff is particularly important in the context of public health emergencies of infectious diseases.

Unlike other disaster events, COVID‐19 is an emerging infectious disease with no evidence‐based and effective treatment options, plus the characteristics of the disease itself. With the high risk of infection and the high intensity of work, health care workers, especially front‐line health care workers, are more prone to symptoms of anxiety, depression, fear, depression, as well as feelings of burnout and psychological stress. Many reports discuss the risk of serious adverse mental health outcomes among health care workers. However, most studies^7^, ^8^, ^9^, ^10^, ^11^, ^12^ focus on cross‐sectional descriptions of psychosocial factors such as population background, job‐related factors, anxiety, and depression. At the beginning of the outbreak, the depression and bad mood of medical staff showed a short‐term effect. Currently, as the COVID‐19 outbreak continues to spread, less has been written about its ongoing impact on the mental health of health care workers. This study investigated and analyzed the mental health status of medical staff in different periods under the public health background of infectious disease outbreak. The purpose of this survey was to conduct a preliminary study of psychological stress in hospital staff in February 2020 and December 2020, to provide information for medical staff to establish sustained psychological crisis intervention in health emergencies and to provide appropriate support for medical staff in health emergencies.

## 2 METHODS

### 2.1 Study design, procedures, and participants

A cross‐sectional study was conducted in February 2020 and December 2020, in which electronic questionnaires were sent to medical workers in several public hospitals in Fuzhou, Fujian province, China. The inclusion criteria: (1) Voluntarily participate in this survey and sign informed consent; (2) This study includes participants between the age range of 18–60 years; (3) Employees who are still working in Grade A public hospitals in Fujian Province. The exclusion criteria: (1) Major physical diseases (such as heart, brain, kidney, etc.) before enrollment; (2) Participants who had a history of mental illness before enrollment (such as schizophrenia, mental retardation, depression, anxiety disorder) were currently taking psychiatric drugs; (3) Currently, we have been dispatched to the severely affected areas.

### 2.2 Ethical considerations

Approval from the Institutional Review Board of the Provincial Hospital (no. 2020‐011) was granted. The purpose, benefits, and uses of the study were explained, confidentiality was assured to all participants, and informed consent was obtained.

### 2.3 Questionnaires and instruments

Questionnaires and instruments consisted of three parts, as described in the following subsections.

#### 2.3.1 Demographic characteristics

In each hospital department, job title, ethnicity, gender, age, occupation, highest educational background, marital status, and self‐rated physical health status were collected.

#### 2.3.2 Mental health assessment

Two scales were used to assess the mental health status of hospital workers. To assess anxiety, the Generalized Anxiety Disorder Scale (GAD‐7) was used, which was originally compiled by Spitzer et al.^13^ for screening anxiety and assessing anxiety severity^14^ for clinical use. The total score is 21 points, which is used to evaluate anxiety level, with 0–4 indicating no anxiety, 5–9 indicating mild anxiety, 10–14 indicating moderate anxiety, and 15–21 indicating severe anxiety.

The Depression Screening Scale (9‐item Patient Health Questionnaire [PHQ‐9]) was used to assess depression. It was originally extracted and used by Kroenke et al.^15^ from the PHQ used for screening for depression and assessing depression severity.^16^ At present, it is widely recommended for use in hospitals and primary health care institutions.^17^ The total score is 27 points, which is used to evaluate the degree of depression, with 0–4 indicating no depression, 5–9 indicating mild depression, 10–14 indicating moderate depression, 15–19 indicating severe depression, and 20–27 indicating very severe depression.

### 2.4 Statistical analysis

The SPSS v.24.0 software package was used for the statistical analysis of data. Mean ± standard deviation was used to describe the distribution of quantitative data. Frequency and composition ratio were used to describe the distribution of qualitative data. The *χ* ^2^ test was used to compare differences in rates between groups, and the Mann–Whitney *U*‐test was used to compare differences in abnormal distribution of quantitative data between groups. All tests were bilateral, the *p*‐value was used to measure the significance if *p* < 0.05 was considered statistically significant.

## 3 RESULTS

**Figure 1:**
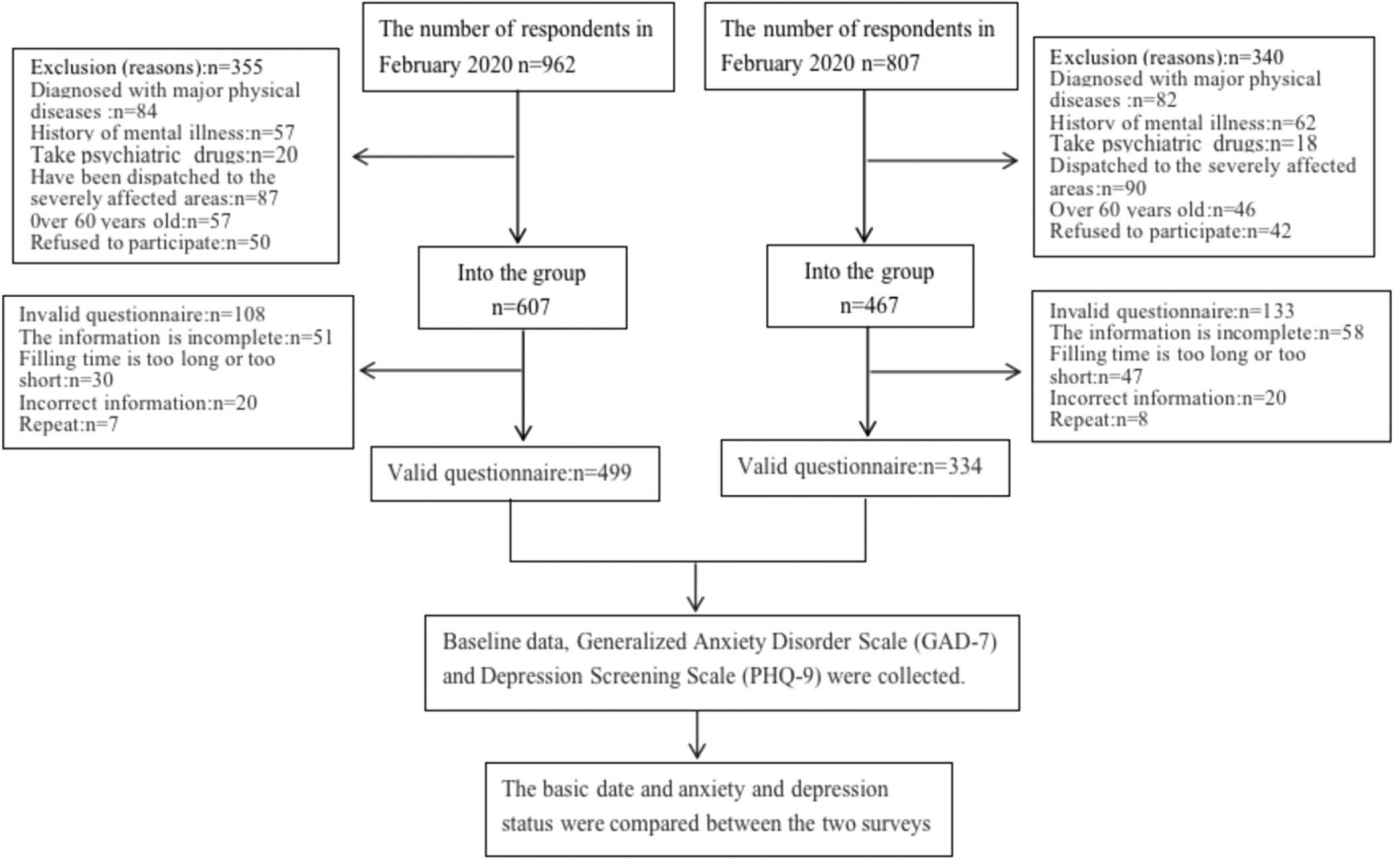
The flow chart of research design

In February and December 2020, the researchers conducted a questionnaire survey using a mobile phone app among health care workers in Fuzhou, Fujian, China. Finally, 499 and 344 valid questionnaires meeting the conditions were collected. The flow chart of the research design is shown in Figure 1.

### 3.1 Population distribution of medical personnel during different periods was different

There were no significant differences among gender, age, marital status, educational background, and physical status of the respondents during the different time periods (*p* > 0.05), but there were significant differences among professional title and occupation (*p* < 0.05), as shown in Table 1.

**Table 1:** Population distribution of medical personnel during different periods

Variable	February 2020	December 2020	*χ* ^2^	*p* Value
Gender			1.193	0.275
Male	111 (33.23%)	148 (29.66%)		
Female	223 (66.77%)	351 (70.34%)		
Age (years)			3.852	0.278
≤30	81 (24.25%)	127 (25.45%)		
31–40	185 (55.39%)	246 (49.3%)		
41–50	63 (18.86%)	114 (22.85%)		
51–60	5 (1.5%)	12 (2.4%)		
Marital status			4.630	0.099
Single	63 (18.86%)	105 (21.04%)		
Married	258 (77.25%)	386 (77.35%)		
Divorced	13 (3.89%)	8 (1.6%)		
Education level			4.664	0.097
College or less	65 (19.46%)	77 (15.43%)		
Undergraduate	199 (59.58%)	289 (57.92%)		
Postgraduate or more	70 (20.96%)	133 (26.65%)		
Physical status			1.168	0.558
General	54 (16.17%)	72 (14.43%)		
In good health	215 (64.37%)	316 (63.33%)		
In excellent health	65 (19.46%)	111 (22.24%)		
Professional title			54.715	<0.001
Junior	96 (28.74%)	211 (42.28%)		
Intermediate	172 (51.5%)	266 (53.31%)		
Senior	66 (19.76%)	22 (4.41%)		
Occupation			40.337	<0.001
Nurse	159 (47.6%)	273 (54.71%)		
Doctor	97 (29.04%)	184 (36.87%)		
Medical technicians	28 (8.38%)	23 (4.61%)		
Other	50 (14.97%)	19 (3.81%)		

*Note*: Data marked by *n* (%) used *χ* ^2^ test.

### 3.2 Differences in anxiety and depression levels in medical staff during different stages of pandemic development

In the early stage of the pandemic in February 2020, 208 professionals exhibited anxiety (41.68%), and there were 152 professionals in December 2020 exhibiting anxiety (45.51%). Anxiety levels showed no significant differences (*χ* ^2^ = 1.193, *p* > 0.05); however, distribution characteristics of anxiety degree in medical staff during different periods were significantly different.

At the beginning of the outbreak in February 2020, the proportion of medical workers with severe anxiety (7.62%) was higher than that in December 2020 (3.59%) (*χ* ^2^ = 21.696, *p* < 0.001). Total anxiety scores in February 2020 were significantly higher than those in December 2020 (*Z* = −16.221, *p* < 0.001), as shown in Table 2.

**Table 2:** Anxiety levels in medical workers during different periods

Variables	Total	Anxiety degree, *n* (%)				Anxiety scores mean (SD)	Positive rate, *n* (%)
		Meaningless	Mild	Moderate	Severe		
December 2020	334	182 (54.49%)	118 (35.33%)	22 (6.59%)	12 (3.59%)	0.53 ± 0.72	152 (45.51%)
February 2020	499	291 (58.32%)	114 (22.85%)	56 (11.22%)	38 (7.62%)	5.34 ± 5.02	208 (41.68%)
Statistics		*χ* ^2^ = 21.696				*Z* = −16.221	*χ* ^2^ = 1.193
*p* Value		<0.001				<0.001	0.275

*Note*: Data marked by mean (SD) used Mann–Whitney *U*‐test. Data marked by *n* (%) used *χ* ^2^ test.

The proportion of people with depression in February 2020 was 22.85%, which was significantly lower than that in December 2020 (49.70%, *χ* ^2^ = 64.663, *p* < 0.001), and the proportion of medical workers with severe depression in December was significantly higher than that in February (*χ* ^2^ = 76.509, *p* < 0.001). At the same time, total depression scores in the early outbreak group (February 2020) were significantly higher than those in December 2020 (*Z* = −7.285, *p* < 0.001), as shown in Table 3.

**Table 3:** Depression levels in medical workers during different periods

Variables	Total	Depression degree, *n* (%)				Depression scores mean (SD)	Positive rate, *n* (%)
		Meaningless	Mild	Moderate	Severe		
December 2020	334	168 (50.3%)	121 (36.23%)	22 (6.59%)	23 (6.89%)	5.72 ± 5.74	166 (49.7%)
February 2020	499	385 (77.15%)	69 (13.83%)	34 (6.81%)	11 (2.2%)	3.05 ± 4.35	114 (22.85%)
Statistics		*χ* ^2^ = 76.509				*Z* = −7.285	*χ* ^2^ = 64.663
*p* Value		<0.001				<0.001	<0.001

*Note*: Data marked by mean (SD) used Mann–Whitney *U*‐test. Data marked by *n* (%) used *χ* ^2^ test.

### 3.3 Differences in anxiety and depression levels of medical workers with different professional titles during different time periods

The severity and total scores of work anxiety in professionals with junior and intermediate titles in February 2020 were significantly higher than those in December 2020 (*p* < 0.05). There were no significant differences in anxiety levels among medical workers with senior professional titles during different time periods (*p* > 0.05), but total anxiety scores decreased (*p* < 0.05), as shown in Table 4.

**Table 4:** Comparison of anxiety levels in medical workers with different professional titles

Professional titles	Variables	Total	Anxiety degree, *n* (%)				Anxiety scores mean (SD)
			Meaningless	Mild	Moderate	Severe	
Junior	December 2020	96	80 (83.33%)	13 (13.54%)	2 (2.08%)	1 (1.04%)	0.2 ± 0.54
	February 2020	211	137 (64.93%)	45 (21.33%)	18 (8.53%)	11 (5.21%)	4.51 ± 4.81
	Statistics		*χ* ^2^ = 12.426				*Z* = −9.776
	*p* Value		0.006				<0.001
Intermediate	December 2020	172	74 (43.02%)	78 (45.35%)	13 (7.56%)	7 (4.07%)	0.65 ± 0.71
	February 2020	266	141 (53.01%)	64 (24.06%)	35 (13.16%)	26 (9.77%)	6.05 ± 5.1
	Statistics		*χ* ^2^ = 24.224				*Z* = −12.546
	*p* Value		<0.001				<0.001
Senior	December	66	13 (59.09%)	5 (22.73%)	3 (13.64%)	1 (4.55%)	0.71 ± 0.84
	February	22	41 (46.59%)	32 (36.36%)	10 (11.36%)	5 (5.68%)	4.68 ± 4.86
	Statistics		‐				*Z* = −3.630
	*p* Value		0.417				<0.001

*Note*: Data marked by mean (SD) used Mann–Whitney *U*‐test. Data marked by *n* (%) used *χ* ^2^ test.

Depression levels and total depression scores of medical workers with junior professional titles in February 2020 were not significantly different from those in December 2020 (*p* > 0.05). The prevalence of depression and total depression score in medical workers with intermediate and senior titles in December 2020 were higher than those in February 2020, and there were significant differences between the two groups (*p* < 0.05), as shown in Table 5.

**Table 5:** Comparison of depression levels in medical workers with different professional titles

Professional titles	Variables	Total	Depression degree, *n* (%)				Depression scores mean (SD)
			Meaningless	Mild	Moderate	Severe	
Junior	December 2020	96	79 (82.29%)	12 (12.5%)	2 (2.08%)	3 (3.12%)	2.35 ± 4.07
	February 2020	211	170 (80.57%)	26 (12.32%)	12 (5.69%)	3 (1.42%)	2.61 ± 4.15
	Statistics		‐				*Z* = −0.387
	*p* Value		0.416				0.699
Intermediate	December 2020	172	69 (40.12%)	79 (45.93%)	12 (6.98%)	12 (6.98%)	6.71 ± 5.32
	February 2020	266	196 (73.68%)	41 (15.41%)	21 (7.89%)	8 (3.01%)	3.46 ± 4.54
	Statistics		*χ* ^2^ = 58.681				*Z* = −6.791
	*p* Value		<0.001				<0.001
Senior	December 2020	66	20 (30.3%)	30 (45.45%)	8 (12.12%)	8 (12.12%)	8.03 ± 6.71
	February 2020	22	19 (86.36%)	2 (9.09%)	1 (4.55%)	0 (0%)	2.36 ± 3.33
	Statistics		‐				*Z* = −4.161
	*p* Value		<0.001				<0.001

*Note*: Data marked by mean (SD) used Mann–Whitney *U*‐test. Data marked by *n* (%) used *χ* ^2^ test.

### 3.4 Comparison of anxiety and depression levels among medical personnel of different professions during different stages of the pandemic

Results showed that anxiety severity and total anxiety scores in doctors, nurses, and other staff members in February 2020 were significantly higher than those in December 2020 (*p* < 0.05). Total anxiety scores of medical technicians during the early stage of the outbreak were significantly higher than those in December 2020 (*p* < 0.05), but there were no significant differences in anxiety severity (*p* > 0.05), as shown in Table 6.

**Table 6:** Comparison of anxiety levels among medical workers of different professions

Profession	Variables	Total	Anxiety degree, *n* (%)				Anxiety scores mean (SD)
			Meaningless	Mild	Moderate	Severe	
Nurse	December 2020	159	116 (72.96%)	30 (18.87%)	8 (5.03%)	5 (3.14%)	0.33 ± 0.69
	February 2020	273	140 (51.28%)	73 (26.74%)	34 (12.45%)	26 (9.52%)	6.03 ± 5.02
	Statistics		*χ* ^2^ = 21.969				*Z* = −13.901
	*p* Value		<0.001				0.000
Doctor	December 2020	97	39 (40.21%)	39 (40.21%)	12 (12.37%)	7 (7.22%)	0.77 ± 0.82
	February 2020	184	125 (67.93%)	33 (17.93%)	16 (8.7%)	10 (5.43%)	4.33 ± 4.82
	Statistics		*χ* ^2^ = 21.858				*Z* = −6.198
	*p* Value		<0.001				<0.001
Medical technicians	December 2020	28	19 (67.86%)	8 (28.57%)	1 (3.57%)	0 (0%)	0.25 ± 0.44
	February 2020	23	14 (60.87%)	5 (21.74%)	2 (8.7%)	2 (8.7%)	5.35 ± 5.77
	Statistics		‐				*Z* = −4.508
	*p* Value		*χ* ^2^ = 0.438				<0.001
Other	December 2020	50	8 (16%)	41 (82%)	1 (2%)	0 (0%)	0.86 ± 0.4
	February 2020	19	12 (63.16%)	3 (15.79%)	4 (21.05%)	0 (0%)	5.21 ± 4.64
	Statistics		‐				*Z* = −4.541
	*p* Value		<0.001				<0.001

*Note*: Data marked by mean (SD) used Mann–Whitney *U*‐test. Data marked by *n* (%) used *χ* ^2^ test.

Results showed that compared to February 2020, the proportion of nurses and other staff members suffering from depression was lower in December 2020, but total depression scores were higher (*p* < 0.05). Also, total depression scores increased (*p* < 0.05). On the contrary, depression rates and total scores in doctors increased significantly in December 2020 (*p* < 0.05). There were no significant changes in the prevalence of depression and total depression scores among medical technicians in December 2020 (*p* > 0.05), as shown in Table 7.

**Table 7:** Comparison of depression levels among medical workers of different professions

Profession	Variables	Total	Depression degree, *n* (%)				Depression scores mean (SD)
			Meaningless	Mild	Moderate	Severe	
Nurse	December 2020	159	111 (69.81%)	34 (21.38%)	5 (3.14%)	9 (5.66%)	3.92 ± 5.1
	February 2020	273	215 (78.75%)	35 (12.82%)	19 (6.96%)	4 (1.47%)	2.86 ± 4.11
	Statistics		*χ* ^2^ = 14.187				Z = −1.970
	*p* Value		0.003				0.049
Doctor	December 2020	97	33 (34.02%)	39 (40.21%)	12 (12.37%)	13 (13.4%)	7.98 ± 6.94
	February 2020	184	138 (75%)	28 (15.22%)	13 (7.07%)	5 (2.72%)	3.14 ± 4.45
	Statistics		*χ* ^2^ = 47.492				Z = −6.325
	*p* Value		<0.001				<0.001
Medical technicians	December 2020	28	16 (57.14%)	9 (32.14%)	2 (7.14%)	1 (3.57%)	4.18 ± 4.31
	February 2020	23	16 (69.57%)	4 (17.39%)	1 (4.35%)	2 (8.7%)	4.61 ± 6.46
	Statistics		‐				Z = −0.467
	*p* Value		0.613				0.640
Other	December 2020	50	8 (16%)	39 (78%)	3 (6%)	0 (0%)	7.92 ± 2.88
	February 2020	19	16 (84.21%)	2 (10.53%)	1 (5.26%)	0 (0%)	3 ± 3.32
	Statistics		‐				Z = −4.811
	*p* Value		<0.001				<0.001

*Note*: Data marked by mean (SD) used Mann–Whitney *U*‐test. Data marked by *n* (%) used *χ* ^2^ test.

## 4 DISCUSSION

The impact of major public health emergencies on an individual's mental health has been demonstrated in previous studies. Under the influence of COVID‐19, the incidence of stress, anxiety, and depression was 29.6%, 31.9%, and 33.7%, respectively.^18^ In major health events, it is impossible to ignore the psychological stress experienced by rescue workers, who are prone to secondary trauma as a result of long‐term contact with crisis situations.^19^ In accordance with previous studies, the overall prevalence of posttraumatic stress disorder (PTSD) among rescue workers is approximately 10%.^20^ As a result of the long working hours, high intensity, and high risk of infection experienced by rescue workers in public health emergencies, this phenomenon is closely related. COVID‐19, which has lasted over a year, has had a number of uncertain effects on the public and health workers. As far as we are aware, this is the first systematic comparative study of the mental health of health care workers during different phases of the COVID‐19 pandemic. There has been an increase in, depression and anxiety among medical staff with different titles and professions as a result of the epidemic.

According to Zhang^21^ and Fen et al.,^22^ public health emergencies have a detrimental effect on medical workers' mental health and increase anxiety, fear, and depression.^23^, ^24^ They may even cause emotional exhaustion and resignation.^25^ According to epidemiological studies, health workers on the front lines are more likely to experience increased stress during pandemics.^26^ Liu et al.^27^ conducted a cross‐sectional survey of medical workers during COVID‐19, and the results showed that depression (50.7%) and anxiety (44.7%) were prevalent. The mental health of medical workers has been studied at different stages of public emergencies, however, few studies have focused on the changes in their mental health over time.

As compared with February 2020, when the pandemic was in its early stages, the proportion of medical workers suffering from severe anxiety was significantly lower in December 2020. As the pandemic progressed, the proportion of professionals with mild anxiety increased significantly, suggesting a slight decrease in anxiety in medical workers. It is possible that this is a direct result of improvements in awareness of COVID‐19 among medical workers, guarantees of protective materials, and management of the pandemic in China.^28^, ^29^ In addition, studies have demonstrated that anxiety can be effectively relieved by interventional measures such as the establishment of reasonable rest and activity areas, the support of mental health experts, and the development of mental health hotlines.^30^, ^31^, ^32^

There was a significant increase in the number of health care workers with major depression and mild depression in December 2020 compared to February 2020. This indicates that depressive symptoms worsened as the pandemic progressed. There are a number of possible reasons for this. First, COVID‐19 has been present for 1 year, and health care personnel is subjected to high stress and risk, making them more susceptible to depression. According to Taylor et al.,^33^ there is a higher incidence of stress, adverse mental health events, and PTSD among first aiders. Medical staff may be able to maintain and promote their mental health if they can identify and address the extent and source of environmental and psychological stress.^34^, ^35^ Second, some studies have revealed that the pathogenesis of depression has a close relationship to inflammation and immune system mechanisms.^36^, ^37^, ^38^ Additionally, short periods of acute stress activate the sympathetic nervous system, stimulate the thalamus–pituitary–adrenal axis, mobilize immune system peripheral inflammatory cells, and regulate inflammatory mediators. In addition, long periods of chronic stress may weaken the immune system and impair the regulation of peripheral inflammation. This may lead to inflammatory substances entering the central nervous system and causing depressive symptoms. Third, this is closely related to the daily medical duties performed by busy hospital workers who do not initiate psychological assistance on their own initiative. According to Chen et al.,^39^ medical staff rarely seek psychological assistance and some even reject it, despite the presence of mental health problems.

The length of time that medical staff has worked, their professional title, their departmental specialty, and their experience with emergency rescue during the COVID‐19 pandemic are considered to be significant factors affecting mental health.^10^, ^40^, ^41^ During different stages of the pandemic, the study examined a variety of professional characteristics of medical workers, as well as differences between anxiety and depression. According to the study, hospital workers' anxiety levels and their levels of depression increased as the COVID‐19 outbreak continued. Consequently, the emphasis on prevention and control of public mental health varies according to the stages of the pandemic. In addition, different measures should be taken during the formation of public mental health prevention and control policies based on the characteristics of various eras.

During the 3‐year follow‐up study^42^ following the SARS outbreak, 23% of health care workers reported moderate or severe depression symptoms. The mental health of medical personnel was therefore required (and continues to be required) during the COVID‐19 outbreak and during the control period. At the beginning of the epidemic and 10 months later, the study only analyzed the emotional status of some staff members working in public hospitals. Although, we did not investigate their mood before the outbreak of COVID‐19. If they were taking drugs that could affect their mood, and whether they had recently been subjected to any significant stressors. Therefore, there could be other factors affecting their emotional state. We can not completely attribute the mood changes to the epidemic, despite the fact that we adjusted for certain factors in our survey. The purpose of our investigation was to identify public hospitals in Fujian province that were not designated to treat patients who were who are infected with viruses. Due to the different epidemic prevention policies, our province implements strict measures for isolation, diagnosis, diagnosis, and treatment. Hospitals that did not specialize in treating people infected with the virus have a lower risk of direct exposure to the virus for their employees. Consequently, the article may reflect some bias regarding the characteristics of the population.

Considering this to be the main drawback of the paper, we will make up for and improve in future research to better assess the impact of this severe outbreak of COVID‐19 on hospital staff, better research methods must be developed. In addition, respondents with moderate or severe anxiety and depression received online feedback, as well as suggestions from psychological professionals. Due to the limitations of the research content, certain factors that are likely to affect psychological outcomes (e.g., medical personnel with different titles and professions) were not explored, which will be improved in future studies.

## 5 CONCLUSION

After the COVID‐19 outbreak in Fujian, China, the anxiety of the public hospital staff had subsided but depression was higher than it was at the beginning of the epidemic. In addition, there were differences in the emotional state of medical staff in different departments of hospitals.^42^ Consequently, different intervention measures should be developed in collaboration with the hospital's management department and psychological intervention personnel.

## AUTHOR CONTRIBUTIONS

**Xing Miao**: Conceptualization; data curation; formal analysis; investigation; methodology; resources; writing – original draft. **Wei Wang**: Conceptualization; data curation; formal analysis; methodology; writing – original draft. **Yongli Chen**: Data curation; formal analysis; investigation. **Xiufang Huang**: Data curation. **Rehua Wang**: Conceptualization; investigation; methodology; project administration; resources; supervision; validation; visualization; writing – original draft; writing – review & editing.

## CONFLICT OF INTEREST

The authors declare no conflict of interest.

## TRANSPARENCY STATEMENT

The lead author Rehua Wang affirms that this manuscript is an honest, accurate, and transparent account of the study being reported; that no important aspects of the study have been omitted; and that any discrepancies from the study as planned (and, if relevant, registered) have been explained.

## Data Availability

R. W. had full access to all of the data in this study and takes complete responsibility for the integrity of the data and the accuracy of the data analysis. All the data and materials are available, the authors confirm that the data supporting the findings of this study are available within the article [and/or] its supplementary materials.
